# Association between resilience and frailty among Chinese older adults

**DOI:** 10.3389/fpsyt.2022.948958

**Published:** 2022-07-15

**Authors:** Yujie Wang, Yingwei Chen, Jixiang Xu, Hao Chen, Junling Gao

**Affiliations:** ^1^School of Public Health, Fudan University, Shanghai, China; ^2^Collaborative Innovation Cooperative Unit of National Clinical Research Center for Geriatric Diseases, Shanghai, China; ^3^Core Unit of Shanghai Clinical Research Center for Geriatric Diseases, Shanghai, China

**Keywords:** resilience, healthy aging, frailty, older adults, mental health

## Abstract

**Purpose:**

Resilience is a multidimensional concept determining healthy aging, however, there were limited studies examining the association between frailty and resilience in detail. In this study, we aimed to examine the association of frailty with three dimensions of resilience-strength, optimism, and tenacity among Chinese older adults.

**Methods:**

A cross-sectional study was conducted among 10,209 participants who were sampled by three-stage sampling method, from three cities in China from June 2020 to July 2021. The Chinese version of the Connor–Davidson Resilience Scale (CD-RISC) was used to measure resilience's 3 dimensions (strength, optimism and tenacity), which were converted into quartiles for the analysis. Frailty status was measured using the Chinese version of the FRAIL scale, categorized into robustness, pre-frailty and frailty. Multinomial logistic regression was used to examine associations between frailty status with strength, optimism and tenacity.

**Results:**

The overall proportions of robustness, pre-frailty, and frailty were 42.7, 48.7, and 8.6%, respectively. After controlling for sociodemographic characteristics, self-rated health, and health behaviors, compared with older adults with the lowest quartile of strength, older adults with the second quartile (odds ratio, OR = 0.67, 95% CI: 0.57–0.78), third quartile (OR = 0.60, 95% CI: 0.50–0.72), and fourth quartile (OR = 0.58, 95% CI: 0.46–0.73) of strength had lower ORs for pre-frailty, and who also had lower ORs (0.44, 95% CI: 0.33–0.58; 0.42, 95% CI: 0.30–0.59; 0.34, 95% CI: 0.20–0.56, respectively) for frailty. There were no homogeneous associations between optimism and tenacity with frailty status.

**Conclusion:**

Higher strength was associated with lower chance of being pre-frail and frail among Chinese older adults. This finding implies that community-based training programs aiming to enhance psychological resilience, especially strength, may contribute to healthy aging. Future studies should examine the effects of resilience on frailty using longitudinal or experimental study designs in cross-cultural contexts.

## Introduction

Frailty is a state of increased vulnerability to poor resolution of homeostasis after a stressor event (1), such as a minor infection or changes in medication or environment. Frail individuals have a higher risk for disability, falls, cognitive decline, admission to the hospital, and the need for long-term care (2–6). Causes of frailty are complex and are likely to involve not only biomedical but also psychosocial mechanisms (7). A recent systematic review found that personality traits (8) were associated with frailty.

As one of the personality traits, resilience is the dynamic process of effectively negotiating, adapting to, or managing important sources of stress or trauma (9). In later life, resilience is defined as a combination of abilities and characteristics that interact dynamically with stressors to permit an individual to recover, cope successfully, and function above the norm despite high levels of stress or adversity (10, 11). Theoretically speaking, resilient older adults may easily return to a basal state and be less likely to be frail (12). Several studies have shown that high resilience is associated with good mental health (13, 14), active aging (15), healthy behaviors (16) and low inflammation biomarker levels (17) in older adults, all of which are important protective factors against frailty. However, there were limited empirical research to examine the relationship of resilience and frailty. One study in 89 geriatric rehabilitation inpatients (18) and another study in 210 community-dwelling older adults (19) both found that resilience was negatively associated with frailty. There is no relevant study regarding the relationship of resilience and frailty among community-dwelling older adults in China.

People from different backgrounds think differently about the realities of adversity and hard times (20). Resilience may manifest differently in different cultural contexts (10), which may be a reason to explain why there was no uniform definition and measurement of resilience. For example, the 25 items of Connor–Davidson Resilience Scale (CD-RISC) (21) was the widely used scale to measure resilience, however which was constructed different factor structures in different cultural contexts (22). The original CD-RISC (21) consists of five dimensions naming tenacity, tolerance of negative affect, secure relationships, control and spiritual influences, while Yu et al. (20) psychometrically validated the Chinese version of CD-RISC constructing three-factor structure what we adopt herein, namely tenacity, strength and optimism. Tenacity describes an individual's equanimity, promptness, perseverance, and sense of control when facing situations of hardship and challenge. Strength focuses on the individual's capacity of recovering and becoming strong after a setback and past experiences. Optimism reflects the individual's tendency to look on the positive sides of things and trust in one's personal and social resources. Studies in older outpatients (23) and older people with HIV (24) found that optimism was negatively associated with frailty. The three dimensions of resilience may have different effects on frailty, however there was no study to simultaneously examine the relationships of these three dimensions of resilience and frailty. In the current study, we aimed to examine the relationships between three dimensions of resilience and frailty among a large sample of general community-dwelling older adults.

## Materials and methods

### Participants and study design

This was a cross-sectional study conducted among Chinese older adults from June 2020 to July 2021. Adults aged ≥65 years from 42 communities were randomly selected using three-stage sampling mothed. Firstly, considering geographical representation and comprehensive development level, we conveniently selected Shanghai (eastern China), Ordos (northern China), and Panzhihua (western China). Next, 16 communities in Shanghai, 9 communities in Ordos, and 17 communities in Panzhihua were conveniently selected. Finally, at least 200 older adults were randomly chosen from each selected community. Participants were eligible if they were aged ≥65 years old and had no progressive tumors or severe mental disorders. Trained interviewers from each selected community visited participants in their homes or invited them to a community health care center to collect data in face-to-face surveys using a self-administered questionnaire. In total, 11,269 older adults were sampled, 10,653 (94.5%) agreed to participated in the current study. We finally included 10,209 participants in the current study after excluding 444 participants with incomplete questionnaires. The written informed consent was obtained from all participants.

### Measurements

#### Frailty

Frailty was measured using the Chinese version of the FRAIL scale (12), which consists of five items (fatigue, resistance, ambulation, illness, and loss of weight). The instrument is simple to use and has shown very good psychometric properties in terms of validity (25–27). Response options for each item were yes or no and for each item, 0 = no, 1 = yes. FRAIL scores range from 0 to 5 and represent frail (3–5), pre-frail (1–2), and robust (0) status.

#### Resilience

The Connor–Davidson Resilience Scale (CD-RISC) is widely used to measure resilience, which is psychometrically robust and adequate for use in older adults (22). The validated Chinese version of CD-RISC (20) was used in the current study, which measures three dimensions of resilience: (1) tenacity (consisting of 13 items), describing an individual's equanimity, promptness, perseverance, and sense of control when facing situations of hardship and challenge; (2) strength (consisting of 8 items), focusing on the individual's capacity of recovering and becoming strong after a setback and past experiences; (3) optimism (consisting of 4 items), reflecting the individual's tendency to look on the positive sides of things and trust in one's personal and social resources. Previous studies have demonstrated that the Chinese version of CD-RISC was psychometric robustness adequate for continued use in Chinese older adults (28, 29). Participants were asked to respond to each item on a 5-point Likert scale, from 0 (not true at all) to 4 (true all the time). To examine the associations between the three dimensions of resilience and frailty, the scores for tenacity (range 0–52), strength (range 0–32), and optimism (range 0–16) were, respectively, calculated. The higher the score, the higher the level of psychological resilience.

#### Covariates

The following covariates were included in this study based on the literature (30–34): age (5-year categories), gender, educational level (under primary school, primary school, junior high school, senior high school and above), marital status [recoded as married and other (including unmarried, divorced, and widowed)], self-rated health [categorized as excellent, good, general, and poor (including poor and very poor)], smoking (never/former/current), drinking (yes/no), fruit intake [≥200 g daily as sufficient (35)], vegetable intake [≥300 g daily as sufficient (35)], and physical activity [≥150 min moderate intensity physical exercise weekly as regular, <150 min weekly as irregular (36)].

### Statistical analysis

Descriptive statistics are reported using median and interquartile range (IQR) or frequency and percentage. Characteristics of participants with different frail statuses were compared using Cochran–Mantel–Haenszel χ^2^ tests for categorical variables. The Wilcoxon rank-sum test and Kruskal–Wallis tests were used to compare resilience scores among participants with different characteristics. As frail status was an ordered multi-category variable, and a significant test statistic provides evidence that the parallel regression assumption has been violated, then multinomial logistic regression was used to estimate odds ratios (ORs) and 95% confidence intervals (95% CIs) of frail status associated with resilience scores, with robust status as the reference category. Model 1 examined the associations of tenacity, strength, and optimism with frailty without controlling for covariates. Model 2 further examined the associations of tenacity, strength, and optimism with frailty after adjusting for all covariates. The data were analyzed using SAS version 9.4 (SAS Institute, Inc., Cary, NC, USA).

## Results

### Descriptive results of demographic characteristics, frailty, and resilience

As shown in Table 1, among all 10,209 participants, the median age was 72 (IQR: 68–77) years old; over a half were female (52.98%), 46.92% of participants had under elementary school education, and 78.1% were married. Over 40% of participants rated their health status as “good” and 38.9% rated their health status as “general.” The prevalence of current smoking and drinking was 17.3 and 13.9%, respectively. Sufficient vegetable and fruit intake was reported by 48.62 and 21.82% of participants, respectively, and 41.1% reported that they undertook physical activity regularly.

**Table 1:** Characteristics of participants and distribution of frailty (*n*, %).

**Characteristics**	***N*** **(%)**	**Frail status**			χ^2^	***P-*** **value**
		**Robust**	**Pre-frail**	**Frail**		
Total	10,209 (100.00)	4,360 (42.71)	4,970 (48.68)	879 (8.61)		
Age (years)					218.232	<0.001
65~	3,525 (34.53)	1,685 (47.80)	1,642 (46.58)	198 (5.62)		
70~	3,110 (30.46)	1,400 (45.02)	1,492 (47.97)	218 (7.0)		
75~	2,095 (20.52)	798 (38.09)	1,068 (50.98)	229 (10.93)		
80~	1,479 (14.49)	477 (32.25)	768 (51.93)	234 (15.82)		
**Gender**					59.727	<0.001
Male	4,800 (47.02)	2,223 (46.31)	2,241 (46.69)	336 (7.00)		
Female	5,409 (52.98)	2,137 (39.51)	2,729 (50.45)	543 (10.04)		
**Education level**					99.363	<0.001
Under primary school	4,790 (46.92)	1,776 (37.08)	2,474 (51.65)	540 (11.27)		
Primary school	3,201 (31.35)	1,534 (47.92)	1,459 (45.58)	208 (6.50)		
Junior high school	1,568 (15.36)	753 (48.02)	735 (46.88)	80 (5.10)		
Senior high school and above	650 (6.37)	297 (45.69)	302 (46.46)	51 (7.85)		
**Marital status**					97.189	<0.001
Unmarried	2,238 (21.92)	814 (36.37)	1,129 (50.45)	295 (13.18)		
Married	7,971 (78.08)	3,546 (44.49)	3,841 (48.19)	584 (7.33)		
**Self-rated health**					793.403	<0.001
Excellent	1,234 (12.09)	587 (47.57)	590 (47.81)	57 (4.62)		
Good	4,156 (40.71)	2,294 (55.20)	1,741 (41.89)	121 (2.91)		
General	3,974 (38.93)	1,341 (33.74)	2,205 (55.49)	428 (10.77)		
Poor	845 (8.28)	138 (16.33)	434 (51.36)	273 (32.31)		
**Smoking**					45.693	<0.001
Never	7,811 (76.51)	3,190 (40.84)	3,904 (49.98)	717 (9.18)		
Former	633 (6.20)	307 (48.50)	268 (42.34)	58 (9.16)		
Current	1,765 (17.29)	863 (48.90)	798 (45.21)	104 (5.89)		
**Drinking**					45.693	<0.001
No	8,793 (86.13)	3,610 (41.06)	4,367 (49.66)	816 (9.28)		
Yes	1,416 (13.87)	750 (52.97)	603 (42.58)	63 (4.45)		
**Vegetable intake**					281.170	<0.001
Not enough	5,245 (51.38)	1,851 (35.29)	2,781 (53.02)	613 (11.69)		
Enough	4,964 (48.62)	2,509 (50.54)	2,189 (44.10)	266 (5.36)		
**Fruit intake**					166.846	<0.001
Not enough	7,981 (78.18)	3,159 (39.58)	4,033 (50.53)	789 (9.89)		
Enough	2,228 (21.82)	1,201 (53.90)	937 (42.06)	90 (4.04)		
**Physical activity**					393.396	<0.001
Irregular	6,014 (58.91)	2,118 (35.22)	3,189 (53.03)	707 (11.76)		
Regular	4,195 (41.09)	2,242 (53.44)	1,781 (42.46)	172 (4.10)		

In total, the proportions of robust, pre-frail, and frail status were 42.7, 48.7, and 8.6%. The prevalence of frailty significantly increased with increasing age (*P* < 0.001) and poorer self-rated health (*P* < 0.001) and with decreasing education level (*P* < 0.001). The prevalence of frailty among women (10.0%) and unmarried older adults (13.2%) was higher than that among their counterparts. The distribution patterns of frailty were significantly different among all health behaviors, with the prevalence of frailty lowest among former smokers (9.2%). Compared with non-drinkers, the prevalence of frailty among drinkers was lower (4.5%). The prevalence of frailty was negatively associated with both vegetable and fruit intake (both *P* < 0.001). Older adults who regularly engaged in physical activity exhibited a low prevalence of frailty (4.1%).

Overall, the median (IQR) for tenacity, strength, and optimism was 28.0 (21.0–36.0), 18.0 (15.0–23.0) and 9.0 (7.0–11.0), respectively. Tenacity was negatively associated with age and self-rated health and positively associated with education level and smoking behavior (all *P* < 0.001). Tenacity among men, married participants, drinkers, those with sufficient vegetable and fruit intake, and those with regular physical activity was higher than that among their counterparts (all *P* < 0.001). The distributions of strength and optimism among covariates followed the same patterns as the distributions of tenacity among the covariates (all *P* < 0.001), as shown in Table 2.

**Table 2:** Median of resilience scores (IQR) by characteristics of participants.

**Characteristics**	**Tenacity (IQR)**	***P-*** **value**	**Strength (IQR)**	***P-*** **value**	**Optimism (IQR)**	***P-*** **value**
**Total**	28.00 (21.00, 36.00)		18.00 (15.00, 23.00)		9.00 (7.00, 11.00)	
Age (years)^a^		<0.001		<0.001		<0.001
65~	30.00 (23.00, 37.00)		19.00 (16.00, 24.00)		9.00 (7.00, 11.00)	
70~	28.00 (22.00, 36.00)		19.00 (15.00, 23.00)		9.00 (7.00, 11.00)	
75~	26.00 (20.00, 34.00)		18.00 (14.00, 22.00)		8.00 (6.00, 10.00)	
80~	26.00 (17.00, 32.00)		16.00 (12.00, 21.00)		8.00 (6.00, 10.00)	
**Gender** ^b^		<0.001		<0.001		<0.001
Male	29.00 (22.00, 37.00)		19.00 (15.00, 23.00)		9.00 (7.00, 11.00)	
Female	26.00 (20.00, 35.00)		18.00 (14.00, 22.00)		8.00 (7.00, 11.00)	
**Education level** ^a^		<0.001		<0.001		<0.001
Under primary school	26.00 (19.00, 33.00)		17.00 (13.00, 22.00)		8.00 (6.00, 10.00)	
Primary school	29.00 (23.00, 36.00)		19.00 (16.00, 23.00)		9.00 (7.00, 11.00)	
Junior high school	32.00 (25.00, 39.00)		20.00 (16.00, 24.00)		9.00 (8.00, 12.00)	
Senior high school and above	34.00 (26.00, 39.00)		22.00 (17.00, 24.00)		10.00 (8.00, 12.00)	
**Marital status** ^b^		<0.001		<0.001		<0.001
Unmarried	26.00 (18.00, 32.00)		17.00 (13.00, 21.00)		8.00 (6.00, 10.00)	
Married	29.00 (22.00, 36.00)		19.00 (15.00, 23.00)		9.00 (7.00, 11.00)	
**Self-rated health** ^a^		<0.001		<0.001		<0.001
Excellent	31.00 (23.00, 38.00)		20.00 (16.00, 24.00)		9.00 (7.00, 11.00)	
Good	30.00 (24.00, 37.00)		20.00 (16.00, 24.00)		9.00 (8.00, 11.00)	
General	26.00 (20.00, 34.00)		17.00 (14.00, 22.00)		8.00 (6.00, 10.00)	
Poor	21.00 (14.00, 28.00)		15.00 (11.00, 19.00)		7.00 (5.00, 9.00)	
**Smoking** ^a^		<0.001		<0.001		<0.001
Never	27.00 (21.00, 36.00)		18.00 (15.00, 23.00)		8.00 (7.00, 11.00)	
Former	27.00 (20.00, 36.00)		18.00 (15.00, 23.00)		8.00 (7.00, 10.00)	
Current	29.00 (23.00, 37.00)		19.00 (16.00, 24.00)		9.00 (7.00, 11.00)	
**Drinking** ^b^		<0.001		<0.001		<0.001
No	27.00 (21.00, 35.00)		18.00 (15.00, 23.00)		8.00 (7.00, 11.00)	
Yes	31.00 (24.50, 37.50)		20.00 (16.00, 24.00)		9.00 (7.00, 11.00)	
**Vegetable intake** ^b^		<0.001		<0.001		<0.001
Not enough	26.00 (19.00, 34.00)		17.00 (13.00, 22.00)		8.00 (6.00, 10.00)	
Enough	30.00 (24.00, 37.00)		20.00 (16.00, 24.00)		9.00 (7.00, 11.00)	
**Fruit intake** ^b^		<0.001		<0.001		<0.001
Not enough	26.00 (20.00, 35.00)		18.00 (14.00, 22.00)		8.00 (6.00, 10.00)	
Enough	32.00 (26.00, 38.00)		20.00 (17.00, 24.00)		10.00 (8.00, 12.00)	
**Physical activity** ^b^		<0.001		<0.001		<0.001
Irregular	26.00 (20.00, 35.00)		17.00 (14.00, 22.00)		8.00 (6.00, 10.00)	
Regular	30.00 (23.00, 37.00)		19.00 (16.00, 24.00)		9.00 (7.00, 11.00)	

a *Kruskal-Wallis test*;

b *Wilcoxon rank-sum test; IQR, inter quartile range*.

### Multivariate analysis for associations between frailty and resilience

The scores of three dimensions of resilience were divided into quartiles to examine their associations with frailty. The results of multinomial logistic regression were presented in Table 3. In terms of the association of resilience with pre-frailty, higher levels of strength and optimism were associated with lower ORs of pre-frailty whereas tenacity was not associated with pre-frailty without adjusting for covariates (Model 1). The ORs (1.26, 95% CI: 1.01–1.59) of pre-frailty among older adults with the highest quartile of tenacity were higher than those among older adults with the lowest quartile of tenacity. The ORs (0.77, 95% CI: 0.68–0.87) of pre-frailty among older adults with the second quartile of optimism were lower than their counterparts with the lowest quartile of optimism. Older adults in the second (0.77, 95% CI: 0.68–0.87), third (0.77, 95% CI: 0.68–0.87), and fourth (0.77, 95% CI: 0.68–0.87) quartiles of strength had lower ORs of pre-frailty compared with their counterparts in the lowest quartile of optimism, after adjusting for covariates (Model 2).

**Table 3:** Multinomial logistic regression analyses of risk factors associated with frailty.

**Variable**	**Model 1**				**Model 2**			
	**Pre-frail**		**Frail**		**Pre-frail**		**Frail**	
	**OR (95% Cl)**	***P-*value**	**OR (95% Cl)**	***P-*value**	**OR (95% Cl)**	***P-*value**	**OR (95% Cl)**	***P-*value**
**Tenacity**								
First quartile	1		1		1		1	
Second quartile	0.94 (0.81–1.10)	0.440	0.70 (0.56–0.89)	0.003	0.93 (0.80–1.09)	0.385	0.77 (0.60–0.99)	0.044
Third quartile	1.01 (0.84–1.21)	0.915	0.67 (0.48–0.93)	0.016	1.02 (0.84–1.23)	0.857	0.81 (0.57–1.15)	0.234
Fourth quartile	1.15 (0.92–1.44)	0.208	0.55 (0.35–0.87)	0.010	1.26 (1.01–1.59)	0.045	0.82 (0.50–1.33)	0.412
**Strength**								
First quartile	1		1		1		1	
Second quartile	0.55 (0.47–0.64)	<0.001	0.30 (0.23–0.39)	<0.001	0.67 (0.57–0.78)	<0.001	0.44 (0.33–0.58)	<0.001
Third quartile	0.48 (0.40–0.57)	<0.001	0.28 (0.20–0.38)	<0.001	0.60 (0.50–0.72)	<0.001	0.42 (0.30–0.59)	<0.001
Fourth quartile	0.44 (0.36–0.56)	<0.001	0.20 (0.12–0.31)	<0.001	0.58 (0.46–0.73)	<0.001	0.34 (0.20–0.56)	<0.001
**Optimism**								
First quartile	1		1		1		1	
Second quartile	0.75 (0.66–0.84)	<0.001	0.75 (0.61–0.93)	0.008	0.77 (0.68–0.87)	<0.001	0.85 (0.68–1.07)	0.167
Third quartile	0.79 (0.68–0.92)	0.003	0.72 (0.53–0.96)	0.026	0.87 (0.74–1.01)	0.072	0.92 (0.67–1.25)	0.581
Fourth quartile	0.80 (0.67–0.95)	0.010	0.79 (0.55–1.14)	0.205	0.86 (0.72–1.03)	0.110	0.98 (0.67–1.44)	0.907

*The covariates controlled by model 2 include: age, gender, education level, marital status, self-rated health, smoking, drinking, vegetable intake, fruit intake, and physical activity*.

In terms of the association of resilience with frailty, higher levels of tenacity, strength, and optimism were associated with lower ORs of frailty, without adjusting for covariates (Model 1). After adjusting for covariates, optimism was not associated with frailty; however, ORs (0.77, 95% CI: 0.60–0.99) of frailty among older adults with the second quartile of tenacity were lower than those among older adults with the lowest quartile of tenacity. Older adults in the second (0.44, 95% CI: 0.33–0.58), third (0.42, 95% CI: 0.30–0.59), and fourth (0.34, 95% CI: 0.20–0.56) quartiles of strength had lower ORs of frailty compared with their counterparts in the lowest quartile of optimism (Model 2).

## Discussion

As a concept in psychology, resilience is a positive factor in successful aging that indicates less frailty in older adults (37). To the best of our knowledge, this was the first study to explore the associations of frailty status and three dimensions of resilience separately among community-dwelling Chinese older adults. Our findings indicated that older adults with higher strength levels were less likely to be in pre-frailty or frailty, but tenacity and optimism was not homogeneously associated with frail status. There was heterogeneity in the association between frailty and resilience dimensions. Our finding was partly consistent with previous finding that resilience was negatively associated with frailty among inpatients (18), outpatients with cirrhosis (38), and community-dwelling older adults (19). These findings imply that training programs to enhance psychological resilience, especially strength, may prevent frailty among older adults.

Strength may enable older adults to cope with stressors better during aging via the following pathways to prevent frailty. First, high levels of strength may promote better mental health or a faster recovery process from poor mental health (39–42). For example, a recent study among nursing home Chinese older adults indicated that resilience could mediate the association between loneliness and frailty (43). Next, high strength levels may be associated with health-promoting behaviors, such as a healthy diet, physical activity, and personal accountability in health care (44). Additionally, high levels of strength are associated with satisfying social relationships (45), functional status (46), and positive coping strategies (37).

It should be noted that tenacity and optimism was not homogeneously associated with pre-frailty and frailty after controlling for covariates. Tenacity describes an individual's equanimity, promptness, perseverance, and sense of control when facing situations of hardship and challenge, so the older adults long-time facing challenges of pre-frailty may become more tenacious. Additionally, a previous study found that optimism was associated with frailty among older people with HIV but not among old people without HIV (24) and older outpatients in clinical setting (23). These findings might indicate there exist complex relationships between resilience and frailty among older adults, which warrants exploration in detail in future research.

This study had several limitations that opened avenues for further research. First, the interview and measurement were generated at a cross-sectional level; thus, we cannot infer a causal relationship between resilience and frailty, such as the conflict finding about older adults with highest quartile of tenacity were more likely to be pre-frail. Second, despite the numerous items on the CD-RISC and covariates measured among a large sample in the current study, other potential confounders, and contextual factors were still absent. Thirdly, we examined the associations between resilience and frailty after controlling for covariates, however those covariates may mediate the associations between resilience and frailty, which needs deep analyses in the future. We suggest the inclusion of additional confounders in future studies examining the effects of resilience on frailty using a longitudinal or experimental study design.

## Conclusion

Our findings extended the limited evidence on the association between resilience and frailty among a large population sample, confirming that higher levels of strength were associated with lower chance being frailty among Chinese older adults. This also implied that community-based programs, such as resistance training (47), positive psychiatry interventions incorporating savoring, gratitude, and engagement in value-based activities (48), and aging-friendly community initiatives (49) to enhance psychological resilience, especially strength, may improve healthy aging.

## Data availability statement

The raw data supporting the conclusions of this article will be made available by the authors, without undue reservation.

## Ethics statement

The studies involving human participants were reviewed and approved by the Research Ethics Committee of the Medical Research at the School of Public Health, Fudan University. The patients/participants provided their written informed consent to participate in this study.

## Author contributions

JG designed the study, obtained the data, and organized the manuscript. YW and YC undertook the analysis supervised by JG and wrote the manuscript. HC, YC, JX, and YW performed the survey. All authors read the final manuscript and agreed with the text.

## Funding

This work was supported by the National Key R&D Program of China (Grant Nos. 2018YFC2002000 and 2018YFC2002001) and the National Natural Science Foundation (Grant No. 82173634).

## Conflict of interest

The authors declare that the research was conducted in the absence of any commercial or financial relationships that could be construed as a potential conflict of interest.

## Publisher's note

All claims expressed in this article are solely those of the authors and do not necessarily represent those of their affiliated organizations, or those of the publisher, the editors and the reviewers. Any product that may be evaluated in this article, or claim that may be made by its manufacturer, is not guaranteed or endorsed by the publisher.
